# How Individual and Contextual Factors Affects Antisocial and Delinquent Behaviors: A Comparison between Young Offenders, Adolescents at Risk of Social Exclusion, and a Community Sample

**DOI:** 10.3389/fpsyg.2017.01825

**Published:** 2017-10-20

**Authors:** Silvia Duran-Bonavila, Andreu Vigil-Colet, Sandra Cosi, Fabia Morales-Vives

**Affiliations:** Psychology, Facultat de Ciencies de l'Educació i Psicologia, Universitat Rovira i Virgili, Tarragona, Spain

**Keywords:** juvenile offenders, social exclusion, personality, intelligence, risk factors

## Abstract

The problems associated with violence during adolescence have been on the rise in recent decades. Many studies have focused only on environmental causes or individual causes of violence, although a combination of both variables would seem to be the best option for prediction. The current study aims to assess the relevance of individual characteristics (personality traits, intelligence, and historical and clinical factors linked to the risk of violence), contextual risk factors and protective factors in explaining antisocial and delinquent behaviors in adolescence by comparing three different samples: a community sample, a sample at risk of social exclusion, and a sample of juvenile offenders. The results show that the samples at risk of social exclusion and the sample of juvenile offenders have a very similar profile in terms of personality traits and intelligence, although they differ from the community sample. However, these two samples do differ in such contextual variables as peer delinquency, poor parental management, community disorganization, or early caregiver disruption.

## Introduction

For several decades now society has been concerned about the high rates of aggressive, violent, and antisocial behaviors in adolescents. For this reason, a great deal of research has focused on determining which variables promote these behaviors or protect against them so that efficient prevention programs can be designed and appropriate interventions implemented. A variety of individual and contextual factors have been shown to be related to these behaviors in adolescence. With regard to individual variables, personality traits such as sensation seeking and impulsivity (e.g., Peach and Gaultney, 2013; Mann et al., 2015, 2017) have been found to be robust predictors of antisocial and criminal behavior (Jones et al., 2011). The meta-analytic review by Jones et al. (2011) of the Big Five personality model has shown show that the most important traits for the prediction of aggressiveness and antisocial behavior are Agreeableness, Conscientiousness, and Neuroticism, with Agreeableness being the most important variable. More specifically, individuals with aggressive and antisocial behavior tend to have lower levels of Agreeableness and Conscientiousness and higher levels of Neuroticism. Other studies have also found higher levels of Extraversion and lower levels of Openness to experience in adolescents with antisocial behavior (Rushton and Chrisjohn, 1981; John et al., 1994; Jin et al., 2016).

Other studies have taken an interest in context and the social environment. Some of these focus specifically on the family, and show that poor parenting is an important predictor for antisocial and criminal behavior in young people (Racz and McMahon, 2011). The authoritarian and permissive parental styles are most related to aggression and delinquency (Palacios, 1999). Authoritarianism is a restrictive style that uses heavy discipline and punishments to control behavior. However, permissive parenting involves indulgent parents who do not exercise enough discipline, and are more responsive than demanding. Parental support and parental knowledge are also important variables. In fact, the study carried out by Cutrín et al. (2017) suggests that parental support is indirectly related to antisocial behavior through parental knowledge. Therefore, a high level of parental support, without parental knowledge, does not reduce antisocial behavior. In fact, it may have the opposite effect and encourage antisocial manifestations because these high levels of parental support alone may be perceived by young people as a reinforcement of their behavior or as permissiveness or ignorance of their misbehavior. Other adults also play an important role in the development of children and adolescents, especially the teachers. In fact, those students with less positive feelings toward and reliance on their teachers tend to display fewer prosocial behaviors, more conduct problems and more hyperactivity/inattention (Longobardi et al., 2016b). However, students who describe the relationship with the teacher as marked by warmth and closeness tend to show more attitudes of openness toward others and prosociality (Longobardi et al., 2016a). Therefore, there is a relationship between conflict in the student-teacher relationship and the manifestation of behavioral or conduct problems (Longobardi et al., 2016a).

Social background and socioeconomic level also have to be taken into account because several studies show that children or adolescents with higher levels of physical aggression are more likely to belong to families with low incomes and with mothers who have a low educational level and dysfunctional parental styles (Haapasalo and Tremblay, 1994; Côté et al., 2006). Although a low socioeconomic level is a predictor of physical aggression, it is not a predictor of indirect aggression (Spieker et al., 2012), which is relational aggression that involves harming a target by rejection, damaging his/her social position (e.g., gossiping or rumor spreading) or manipulating peer relationships (e.g., targets are excluded from activities, ostracized, and have their friendships sabotaged). Several longitudinal studies have also linked some issues related to low socioeconomic status (such as low family income, living in subsidized housing, or low parental education) with delinquency (Elliott and Ageton, 1980; Bjerk, 2007). Furthermore, being raised in a family with a low socioeconomic level usually involves being exposed to other risk factors, which may increase the risk of engaging in antisocial behaviors, such as belonging to a dysfunctional family (Pagani et al., 2010) or living in disadvantaged neighborhoods (Elliott et al., 2015).

The present study compares three different samples of adolescents, one of which has presented antisocial and criminal behavior with legal consequences. In particular, a sample of juvenile offenders is compared with a community sample and a sample at risk of social exclusion. This comparison is made in an attempt to determine the profile of personality, abilities, characteristics of the environment, etc. that differentiate the sample of juvenile offenders from the other samples, since identifying these characteristics may help us to understand why some adolescents commit crimes and engage in antisocial behavior that have a negative effect on other people. Taking into account that a combination of individual and contextual factors seems to be the best way of predicting antisocial behavior (Sampson and Lauritsen, 1994), although few studies include both sources of variables, in the current study we have assessed individual characteristics (personality traits, intellectual abilities, and historical and clinical factors linked to risk of violence) and contextual risk factors (such as family factors, peer delinquency, or community disorganization), as well as protective factors linked to violence.

It should be taken into account that social exclusion is a complex concept that encompasses a wide range of situations and processes related to poverty, deprivation, and hardship, but it can also encompass a wide range of categories of excluded people and places of exclusion (Peace, 2001). Therefore, social exclusion is not a synonym of poverty, because it also involves other disadvantages and marginalized statuses, whereby the individual is effectively prevented from fully participating in modern society, without full access to the various opportunities and resources available to the rest of society (internet, employment, housing, health care, political participation, etc.). The fact is that adolescents at risk of social exclusion have many risk factors in common with juvenile offenders so, although they may share certain personality characteristics and abilities, the personal or environmental variables that differentiate the two kinds of adolescent are of great interest.

So, the main objectives of this research are (a) to analyze the extent to which the personality and abilities of a community sample differ from those of a sample of adolescents at risk of social exclusion and a sample of juvenile offenders, and (b) to determine which variables may be responsible for the differences between adolescents at risk and offenders. Therefore, we expect to find differences between the three samples in several individual and contextual characteristics: for example, lower levels of Conscientiousness, Emotional stability, Agreeableness, and Openness to experience, and higher levels of Extraversion for the juvenile justice sample, and higher levels of impulsivity and aggressiveness, especially physical and proactive aggressiveness. We also expect to find lower levels of intelligence in the sample at risk of social exclusion than in the community sample. In fact, the sample at risk of social exclusion may have had fewer educational opportunities, less stimulation during childhood and an inappropriate family context that may affect learning processes linked to crystallized intelligence. We also expect to find lower levels of intelligence in the sample of juvenile offenders, because the lack of strategies for solving problems, such as verbal abilities, is linked to antisocial behavior (Garaigordobil, 2004). Furthermore, some studies show that there is a significant positive correlation between intelligence and income level (Rowe et al., 1998), and a significant negative correlation between intelligence and delinquency (Farrington and West, 1990). In fact, a low level of intelligence is considered to be a risk factor for antisocial behavior (Levine, 2011) because intelligence plays an important role in psychosocial adaptation. We expect to find more historical risk factors linked to violence in the sample of juvenile offenders: for example, more childhood histories of maltreatment, self-harm or suicide attempts or an early initiation of violence. Finally, we also expect this group to obtain higher levels in some individual/clinical and contextual risk factors related to callousness and antisocial behavior, such as low empathy and remorse, substance-use difficulties, peer delinquency, negative attitudes, and low interest and commitment to school.

## Materials and methods

### Participants

This study involved the participation of 1,041 young people between 12 and 21 years old from the provinces of Tarragona, Barcelona, and Lleida (Spain). However, the data of 105 individuals was removed because they had not responded to the questionnaires correctly (several items unanswered, personality questionnaires unfinished or questionnaires clearly answered at random), so finally the three samples contained 936 individuals with the following characteristics:

1. The community sample was collected in five high schools from Tarragona (Spain). The sample was made up of 528 individuals (51.5% boys), of whom 78.6% were born in Spain and 21.4% elsewhere (especially Arabic, Latin American, and Eastern European countries).
2. The sample at risk of social exclusion was recruited in shared education units^1^ (UEC, 64 individuals), open centers (CA, 159 individuals), and training and job programs (PFI programs, 55 individuals). The open centers and shared education units are run by the Network of Social Services and the Education Network, respectively, and are designed to assist children and adolescents at risk of social exclusion. This sample is made up of 278 individuals (62.2% boys), of whom 56.7% were born in Spain, and 43.3% elsewhere (especially in Arabic, Latin American, and Eastern European countries).
3. The sample of juvenile offenders was recruited from open intervention programs and juvenile offenders' education centers^2^. This sample is made up of 130 individuals (88.5% boys), of whom 51.5% were born in Spain, and 48.5% elsewhere (especially in Arabic and Latin American countries).

We found significant differences between the three groups in sex (χ^2^ = 60.2, *p* < 0.01) and country of origin (χ^2^ = 154.7, *p* < 0.01). For this reason, we matched the individuals of the three samples by sex and country of origin (national or foreign). Therefore, each sample is finally made up of 120 individuals (89% boys, 51% of whom are from Spain), with the following characteristics:

1. The members of the community sample were between 11 and 22 years old (*M* = 16.2, *S.D*. = 2.6). The Hollingshead Four-Factor Index of Socioeconomic Status ranged between 8 and 62 (*M* = 31.1, *S.D*. = 15.1).
2. The members of the sample at risk of social exclusion were between 12 and 21 years old (*M* = 15.3, *S.D*. = 1.5). The Hollingshead Four-Factor Index of Socioeconomic Status ranged between 8 and 41 (*M* = 17.3, *S.D*. = 8.3).
3. The members of the sample of juvenile offenders were between 14 and 21 years old (*M* =, 17.6, *S.D*. = 1.2). The Hollingshead Four-Factor Index of Socioeconomic Status ranged between 8 and 61 (*M* = 14.9, *S.D*. = 10.1).

### Measures

#### The indirect-direct aggression questionnaire (I-DAQ; Ruiz-Pamies et al., 2014)

This test contains three subscales: physical aggression (PA), verbal aggression (VA), and indirect aggression (IA). It also provides scores for overall aggression. The test was developed using a method that controls social desirability and acquiescence, which can have a considerable effect on the scores and factor structure of aggressive behavior self-reports. The subscales have appropriate factorial reliabilities in this sample: *r*_θθ_ = 0.83, *r*_θθ_ = 0.78, and *r*_θθ_ = 0.81 for PA, VA, and IA, respectively.

#### Proactive/reactive aggression questionnaire for teachers (PRA-t; Cosi et al., 2009)

This questionnaire assesses aggressiveness in children and adolescents, and it is reported by teachers. It contains eight items: four on Proactive aggression and four on Reactive aggression. The internal consistencies of the subscales are adequate: α = 0.91 for the total score, α = 0.90 for the reactive scale and α = 0.91 for the proactive scale. The questionnaire was answered only by the at-risk and delinquent samples.

#### Barratt impulsiveness scale-11 for children (BIS-11c; Chahin et al., 2010)

This questionnaire is a modified Spanish version of Barratt's BIS-11 (Barratt, 1985) adapted for children and adolescents. It contains 26 items that measure three components of impulsiveness: Motor Impulsiveness (MI), Cognitive Impulsiveness (CI), and Non-Planning Impulsiveness (N-PI). The internal consistencies of the subscales are: α = 0.68 for CI, α = 0.73 for N-PF, and α = 0.80 for MI.

#### Overall personality assessment scale (OPERAS; Vigil-Colet et al., 2013)

This scale is based on the five-factor model of personality, and it measures the following traits: Extraversion (EX), Emotional Stability (ES), Conscientiousness (CO), Agreeableness (AG), and Openness to Experience (OE). The questionnaire contains 40 items, and the subscales have appropriate factorial reliabilities: *r*_θθ_ = 0.86 for EX, *r*_θθ_ = 0.86 for ES, *r*_θθ_ = 0.77 for CO, *r*_θθ_ = 0.71 for AG, and *r*_θθ_ = 0.81 for OE.

#### Thurstone's primary mental abilities (PMA; Cordero et al., 1999)

The subscales of Thurstone's test are: Verbal (PMA-V), Spatial (PMA-S), Numerical (PMA-N), Reasoning (PMA-R), and Word Fluency (PMA-WF). This test comprises scales of fluid and crystallized intelligence.

#### Raven's progressive matrices test (Raven et al., 1996)

This test may be considered a measure of fluid intelligence that is free of cultural bias. We use the Standard scale, which consists of 60 problems.

#### Information scale of the wechsler intelligence scale for children (WISC-IV; Wechsler, 2003)

The Information scale of the WISC-IV is an indicator of crystallized intelligence and assesses general cultural knowledge, long-term memory, and acquired facts.

#### Hollingshead four-factor index of socioeconomic status (Hollingshead, 1975)

This index is widely used to assess the social status of an individual's family, taking into account four domains: education, occupation, sex, and marital status. In the case of single-parent families, this index is calculated from the family member or referent who lives with the minor.

#### Structured assessment of violence risk in youth (SAVRY; Bartel et al., 2000)

This survey is used to estimate the risk of violence among adolescents aged 12–18. It consists of 30 items: 24 risk items and 6 items to assess protective factors. The risk items are divided into three domains: Historical factors (for example, early initiation of violence), Social/Contextual factors (for example, poor parental management), and Individual/Clinical factors (for example, risk taking and impulsivity). The risk items are rated on a three-point scale (low, moderate, high), and the protective factors are rated as either present or absent. This questionnaire was answered only in the at-risk and delinquent samples.

### Procedure

This study was carried out in accordance with the recommendations of the Spanish organic law 15/1999 and the Spanish Agency for Data Protection, which regulate the fundamental right to the protection of data. This project and the protocol were approved by the ethical committee of our Faculty. Moreover, we obtained parental written informed consent from all subjects. All parents gave written informed consent in accordance with the Declaration of Helsinki.

The community sample was recruited from five high schools. The questionnaires were administered collectively during regular school hours, by a professional psychologist, and students were guaranteed anonymity and confidentiality. Participation was voluntary. School approval and parental written informed consent were obtained before the study. About 96% of the participants who were invited to participate in the study eventually did so. This sample answered all the questionnaires except SAVRY and APR, because they are not self-report questionnaires.

For the sample at risk of social exclusion, we asked the people in charge of Social Services, Social Welfare, the Local Council, UECs, and FIAP centers for permission. The same procedure was followed for the sample of juvenile offenders, and we sought the permission of the departments and professionals involved. Then, parental written informed consent was obtained for minors, while participants aged 18 years old or over consented by themselves.

In the sample at risk of social exclusion, questionnaires were administered by a professional psychologist in groups of up three people. In the sample of juvenile offenders, tests were administered individually, by the same professional psychologist. Participation was voluntary and students were guaranteed anonymity and confidentiality.

The questionnaires were administered in two sessions, on two different days, to avoid the effects of fatigue due to the considerable number of questionnaires. The first session involved the administration of OPERAS, I-DAQ, BIS-11c, and RAVEN, and the second session involved PMA, WISC-IV, and the Hollingshead Four-Factor Index of Socioeconomic Status. The questionnaires PRA-t and SAVRY were only administered in the sample at risk of social exclusion and the sample of juvenile offenders, because they are hetero-administered questionnaires that have to be completed by professional evaluators who know the characteristics of each individual.

### Data analysis

As has been explained above, before we made the statistical analysis, we matched the individuals of the three samples for sex and country of origin (national or foreign) because the samples were very different (there were more girls and Spanish individuals in the community sample). We also found differences in the socioeconomic level between the three groups (χ^2^ = 196.7, *p* < 0.01), with higher levels in the community sample. For this reason, it has been introduced as a covariant to analyze those variables that are significantly correlated with socioeconomic level. Table 1 shows the variables significantly correlated with socioeconomic level.

**Table 1:** Significant correlations between socioeconomic level (Hollinsghead index) and variables of personality and capacity in the matched sample.

	**Scale**	**Hollinsghead index**
OPERAS	Openness	0.21^**^
I-DAQ	Physical	−0.15^**^
	Verbal	−0.14^**^
	Indirect	−0.25^**^
	Overall scores	−0.25^**^
PMA	PMA-V	0.21^**^
	PMA-R	0.24^**^
	PMA-WF	0.27^**^
	Overall scores	0.25^**^
WISC-IV	Information	0.43^**^
RAVEN	Overall scores	0.28^**^
G factor		0.32^**^

** p < 0.01

As far as statistical analyses were concerned, we performed several Analyses of Covariance (ANCOVA). We used the 1% level of significance to avoid an excessive experimentalwise error rate (EER) as a consequence of the considerable number of significance tests that we conducted. Moreover, taking into account that the ANOVA/ANCOVA analyses are not robust to the lack of homoscedasticity, we performed Levene's Test for Equality of Variances in all the variables used in these analyses. The results show that the assumption of homoscedasticity is fulfilled (*p* > 0.05 in all the scales). We also carried out chi-square tests to determine the differences between the sample at risk of social exclusion and the sample of juvenile offenders in the psychosocial variables assessed by the SAVRY questionnaire.

General intelligence was estimated by computing for each individual their factorial score on the first factor extracted by maximum likelihood using all the intelligence measures. The Kaiser-Meyer-Olkin (KMO) index value was 0.83, which suggests that the correlation matrix is well-suited to factor analysis (see Kaiser and Rice, 1974). Only the first factor had an eigenvalue higher than 1, which explains 44.2% of variance. The loadings ranged between 0.52 and 0.65.

## Results

Table 2 shows the descriptive statistics for the personality measures across the three samples. It also shows the ANOVA/ANCOVA analyses for the comparison of means between the three samples. More specifically, an ANCOVA was carried out with the variables that correlated with the socioeconomic level (Openness to experience and the subscales and total scores of I-DAQ), and an ANOVA with the other variables. As can be seen, significant differences were found for the subscales extraversion, conscientiousness, and openness to experience in the OPERAS test, although the effect size is small for extraversion and conscientiousness, and medium for openness to experience. As can be seen in Table 3, pairwise mean comparisons show that there are significant differences between the community and risk samples for extraversion and openness to experience. There are also significant differences between the community and juvenile offenders samples for extraversion and openness to experience, and significant differences between the juvenile offenders and risk samples for conscientiousness. For the I-DAQ questionnaire, significant differences between the samples were found for physical and indirect aggressiveness, and also for total scores. More specifically, pairwise mean comparisons show that there are significant differences between the community and risk samples for physical aggressiveness, indirect aggressiveness, and total scores. Significant differences were also found between the community and juvenile offenders samples for physical aggressiveness and total scores. With regard to BIS-11c, significant differences were found between the community and risk samples and between the community and juvenile offenders samples for motor impulsiveness.

**Table 2:** Descriptive statistics for the personality measures in the three samples and ANOVA/ANCOVA analyses.

		**Control**		**Risk**		**Justice**		**F**	**η^2^**
**Tests**	**Subscales**	**Mean**	***S.D*.**	**Mean**	***S.D***.	**Mean**	***S.D***.		
	EX	48.4	9.9	51.3	9.7	52.0	8.2	5.18^**^	0.028
	ES	50.4	10.3	51.7	10.9	51.3	8.7	0.48	0.003
OPERAS	CO	44.9	10.6	43.3	10.8	47.7	9.8	5.62^**^	0.031
	AG	50.0	10.4	48.7	10.7	51.8	10.5	2.67	0.015
	OE	44.2	11.2	40.4	9.9	37.7	10.4	8.20^**^	0.046
	PHYS	55.2	12.3	63.7	11.6	63.8	11.8	13.77^**^	0.075
I-DAQ	VER	50.3	9.6	53.2	9.4	53.1	9.7	2.45	0.014
	IND	54.1	9.5	58.2	10.3	56.7	9.2	4.14^**^	0.024
	TOTAL	54.7	10.1	61.8	9.9	61.0	9.3	12.91^**^	0.071
	COG	12.2	2.4	12.5	2.5	13.1	2.5	4.28	0.023
BIS-11c	NON-P	9.2	3.8	9.7	4.3	8.9	4.1	1.39	0.008
	MOT	25.3	5.8	27.4	6.2	27.4	6.3	4.9^**^	0.027

*EX, Extraversion; ES, Emotional stability; CO, Conscientiousness; AG, Agreeableness; OE, Openness to experience; PHYS, Physical aggressiveness; VER, Verbal aggressiveness; IND, Indirect aggressiveness; TOTAL, overall scores in I-DAQ; COG, Cognitive Impulsiveness; NON-P, Non-planning Impulsiveness; MOT, Motor Impulsiveness*.

** *p < 0.01*.

**Table 3:** Pairwise comparisons for the personality measures.

		**Risk vs. Control**		**Justice vs. Control**		**Justice vs. Risk**	
**Tests**	**Subscales**	**Dif**.	**Cohen's *d***	**Dif**.	**Cohen's *d***	**Dif**.	**Cohen's *d***
	EX	2.98^**^	0.30	3.65^**^	0.39	0.68	–
	ES	0.36	–	0.87	–	−0.36	–
OPERAS	CO	−1.65	–	2.81	–	4.46^**^	0.43
	AG	−1.34	–	1.80	–	3.14	–
	OE	−3.83^**^	−0.36	−6.51^**^	−0.60	−2.55	–
	PHYS	8.47^**^	0.71	8.63^**^	0.72	−0.09	–
I-DAQ	VER	2.96	–	3.43	–	0.47	–
	IND	4.10^**^	0.41	2.58	–	1.04	–
	TOTAL	7.09^**^	0.71	6.28^**^	0.65	0.50	–
	COG	0.27	–	0.91	–	0.64	–
BIS-11c	NON-P	0.55	–	0.31	–	0.86	–
	MOT	2.16^**^	0.36	2.13^**^	0.35	−−1.35	–

*Dif., Difference of means; EX, Extraversion; ES, Emotional stability; CO, Conscientiousness; AG, Agreeableness; OE, Openness to experience; PHYS, Physical aggressiveness; VER, Verbal aggressiveness; IND, Indirect aggressiveness; TOTAL, overall scores in I-DAQ; COG, Cognitive Impulsiveness; NON-P, Non-planning Impulsiveness; MOT, Motor Impulsiveness*.

** *p < 0.01*.

Table 4 shows the descriptive statistics for the intelligence measures across the three samples, and it also shows the ANCOVA analyses for the comparison of means between the three samples, controlling for age and socioeconomic level (Hollingshead Four-Factor Index). As Table 4 shows, significant differences were found for two subscales of PMA, Reasoning and Number, and for the overall scores. As can be seen in Table 5, pairwise mean comparisons show that there are significant differences between the community and risk samples for Reasoning, Number, and overall scores. There are also significant differences between the community and juvenile offenders sample for Reasoning and overall scores. There was only one significant difference between the sample at risk of social exclusion and the juvenile offenders sample for Number. As far as the other measures are concerned, significant differences between groups were found for the Information subscale of WISC-IV, the Raven's progressive matrices test and the overall scores on Factor G. More specifically, pairwise mean comparisons show that there are significant differences between the community and risk samples for the Information subscale, Raven and Factor G. Likewise, there are significant differences between the community and juvenile offenders samples for the Information subscale, Raven, and Factor G.

**Table 4:** Descriptive statistics for the intelligence measures in the three samples and ANCOVA analyses.

		**Control**		**Risk**		**Justice**		**F**	**η^2^**
**Tests**	**Subscales**	**Mean**	**S.D**.	**Mean**	**S.D**.	**Mean**	**S.D**.		
	PMA-V	17.3	7.6	15.1	6.8	15.3	6.9	2.51	0.016
	PMA-S	20.8	11.2	19.8	11.5	18.9	10.8	0.63	0.004
PMA	PMA-R	13.0	6.4	8.9	5.4	18.9	10.8	11.12^**^	0.066
	PMA-N	9.8	5.6	6.5	5.7	8.7	5.7	8.38^**^	0.050
	PMA-F	35.0	11.1	32.9	10.4	33.0	10.6	0.97	0.006
	Total	117.7	38.9	99.2	33.7	102.4	37.2	6.01^**^	0.037
WISC-IV	INF	11.9	4.5	9.3	3.8	9.0	4.2	12.98^**^	0.076
RPM	Raven	47.0	6.5	42.2	7.6	40.5	8.8	15.25^**^	0.088
FACTOR G	Total	53.7	8.9	48.0	7.9	48.0	9.0	8.62^**^	0.070

*PMA-V, PMA Verbal Meaning; PMA-S, PMA Space; PMA-R, PMA Reasoning; PMA-N, PMA Number; PMA-F, PMA Word Fluency; INF, WISC-IV Information; RPM, Raven's Progressive Matrices test*.

** *p < 0.01*.

**Table 5:** Pairwise comparisons for the intelligence measures.

		**Risk vs. Control**		**Justice vs. Control**		**Justice vs. Risk**	
**Tests**	**Subscales**	**Dif**.	**Cohen's *d***	**Dif**.	**Cohen's *d***	**Dif**.	**Cohen's *d***
	PMA-V	−2.24	–	−2.06	–	0.18	–
	PMA-S	−1.06	–	−1.94	–	−0.88	–
PMA	PMA-R	−4.02^**^	−0.68	−3.44^**^	−0.56	−0.58	–
	PMA-N	−3.35^**^	−0.59	−1.16	–	2.19^**^	0.39
	PMA-F	−2.08	–	−1.99	–	0.09	–
	Total	−18.47^**^	−0.51	−15.28^**^	−0.40	3.19	–
WISC-IV	INF	−2.62^**^	−0.63	−2.99^**^	−0.69	−0.36	–
RPM	Raven	−4.72^**^	−0.67	−6.46^**^	−0.84	−1.75	–
FACTOR G	Total	−5.74^**^	−0.68	−5.66^**^	−0.63	0.08	–

*Dif., Difference of means; PMA-V, PMA Verbal Meaning; PMA-S, PMA Space; PMA-R, PMA Reasoning; PMA-N, PMA Number; PMA-F, PMA Word Fluency; INF, WISC-IV Information; RPM, Raven's Progressive Matrices test*.

** *p < 0.01*.

These results show that the juvenile offenders sample and the sample at risk of social exclusion have a very similar profile in terms of personality traits and intelligence variables. However, they differ in other variables (see below). Table 6 shows the results of both samples for the PRA-t questionnaire. As can be seen, there are no significant differences for reactive aggression between the two samples. However, there are significant differences in proactive aggression, the scores being higher for the juvenile offenders sample.

**Table 6:** Differences in proactive and reactive aggression between the sample of juvenile justice and the sample at risk of social exclusion.

		**Risk**	**Justice**		
**Tests**	**Subscales**	**Mean**	**Mean**	***t***	***d***
PRA-t	Proactive	5.8	6.7	2.96^**^	0.35
	Reactive	9.4	9.8	1.3	–

** *p < 0.01*.

Table 7 shows the results for both samples on the risk factors of the SAVRY questionnaire. As can be seen in the table, there are significant differences in most of the historical risk factors. In fact, a higher percentage of juvenile offenders have moderate or high levels of history of violence, history of non-violent offending, and early initiation of violence. In terms of family history, a higher percentage of juvenile offenders have high levels of early caregiver disruption and childhood history of maltreatment. A higher percentage of juvenile offenders also have a history of self-harm or suicide attempts as well as poor school achievement. Likewise, there are significant differences in most individual/clinical risk factors. In fact, a higher percentage of juvenile offenders have high or moderate levels of substance-use difficulties, low interest in or commitment to school and negative attitudes. There are also significant differences between both groups in the risk factor low empathy/remorse, with a higher number of youth offenders showing low empathy and remorse. However, a higher number of adolescents from the at-risk sample have attention deficit/hyperactivity difficulties. Finally, there are significant differences between both groups in four social/contextual risk factors: peer delinquency; stress and poor coping; poor parental management; and community disorganization. Juvenile offenders have higher or moderate levels.

**Table 7:** Percentage of adolescents with low, moderate, and high levels of risk factors in SAVRY, in juvenile justice, and risk samples.

			**Risk**	**Justice**	**χ^2^**	**Contingency coefficient**
Historical risk factors	History of violence	Low (%)	79.06	33.33	90.3^**^	0.45^**^
		Moderate (%)	17.95	32.50		
		High (%)	2.99	34.17		
	History of non-violent offending	Low (%)	81.78	35.83	83.9^**^	0.43^**^
		Moderate (%)	16.10	40.83		
		High (%)	2.12	23.33		
	Early initiation of violence	Low (%)	86.09	56.67	39.0^**^	0.32^**^
		Moderate (%)	11.30	30.00		
		High (%)	2.61	13.33		
	History of self-harm or suicide attempts	Low (%)	88.84	83.33	9.6^**^	0.16^**^
		Moderate (%)	10.30	10.83		
		High (%)	0.86	5.83		
	Exposure to violence in the home	Low (%)	62.21	61.67	5.9	0.13
		Moderate (%)	26.27	18.33		
		High (%)	11.52	20.00		
	Childhood history of maltreatment	Low (%)	73.30	60.83	15.2^**^	0.21^**^
		Moderate (%)	23.53	25.00		
		High (%)	3.17	14.17		
	Parental/Caregiver criminality	Low (%)	80.29	66.67	8.2	0.16
		Moderate (%)	12.50	18.33		
		High (%)	7.21	15.00		
	Early caregiver disruption	Low (%)	74.89	68.33	15.6^**^	0.21^**^
		Moderate (%)	22.08	17.50		
		High (%)	3.03	14.17		
	Poor school achievement	Low (%)	29.91	11.67	19.5^**^	0.23^**^
		Moderate (%)	33.76	30.83		
		High (%)	36.32	57.50		
Individual/clinical risk factors	Negative attitudes	Low (%)	59.75	31.67	25.6^**^	0.26^**^
		Moderate (%)	33.90	55.00		
		High (%)	6.36	13.33		
	Risk taking	Low (%)	42.98	34.17	2.6	0.09
		Moderate (%)	40.85	46.67		
		High (%)	16.17	19.17		
	Substance-use difficulties	Low (%)	70.21	45.83	22.8^**^	0.25^**^
		Moderate (%)	21.70	32.50		
		High (%)	8.09	21.67		
	Anger management problems	Low (%)	47.03	44.17	0.6	0.05
		Moderate (%)	40.68	40.83		
		High (%)	12.29	15.00		
	Low empathy/Remorse	Low (%)	59.83	45.83	13.6^**^	0.19^**^
		Moderate (%)	34.19	36.67		
		High (%)	5.98	17.50		
	Attention deficit/Hyperactivity difficulties	Low (%)	46.41	62.50	9.8^**^	0.16^**^
		Moderate (%)	40.08	24.17		
		High (%)	13.50	13.33		
	Poor compliance	Low (%)	63.56	51.67	5.4	0.12
		Moderate (%)	28.81	40.83		
		High (%)	7.63	7.50		
	Low interest/Commitment to school	Low (%)	61.97	36.44	20.6^**^	0.24^**^
		Moderate (%)	26.50	43.22		
		High (%)	11.54	20.34		
Social/contextual risk factors	Peer delinquency	Low (%)	59.48	17.50	94.4^**^	0.46^**^
		Moderate (%)	36.21	41.67		
		High (%)	4.31	40.83		
	Peer rejection	Low (%)	69.07	70.83	0.2	0.03
		Moderate (%)	26.69	25.83		
		High (%)	4.24	3.33		
	Stress and poor coping	Low (%)	53.28	37.50	9.4^**^	0.16^**^
		Moderate (%)	29.69	45.00		
		High (%)	17.03	17.50		
	Poor parental management	Low (%)	46.75	20.17	25.3^**^	0.26^**^
		Moderate (%)	36.80	48.74		
		High (%)	16.45	31.09		
	Lack of personal/social support	Low (%)	48.21	44.17	0.6	0.04
		Moderate (%)	41.07	43.33		
		High (%)	10.71	12.50		
	Community disorganization	Risk	44.68	34.75	11.7^**^	0.18^**^
		Low (%)	40.85	35.59		
		Moderate (%)	14.47	29.66		

** *p < 0.01*.

Finally, we assessed differences between both groups in the protective factors of SAVRY. As can be seen in Table 8, significant differences were found for the following protective factors: prosocial involvement, strong attachments and bonds, and resilient personality traits. More specifically, the juvenile offenders sample has higher percentages of absence of these protective factors.

**Table 8:** Percentage of adolescents with low, moderate, and high levels of protective factors, in juvenile justice, and risk samples.

**Protective factors**		**Risk**	**Justice**	**χ^2^**	**Contingency coefficient**
Prosocial involvement	Present (%)	68.22	38.79	25.7^**^	0.27^**^
	Absent (%)	31.78	61.21		
Strong social support	Present (%)	58.44	63.87	0.9	0.33
	Absent (%)	41.56	36.13		
Strong attachments and bonds	Present (%)	77.23	55.46	17.4^**^	0.22^**^
	Absent (%)	21.37	44.54		
Positive attitude toward intervention and authority	Present (%)	78.63	74.79	0.7	0.04
	Absent (%)	21.37	25.21		
Strong commitment to school or work	Present (%)	58.62	47.90	3.7	0.10
	Absent (%)	41.38	52.10		
Resilient personality traits	Present (%)	64.56	37.82	23.0^**^	0.25^**^
	Absent (%)	35.44	62.18		

** *p < 0.01*.

## Discussion

The main aim of this study was to determine which variables may facilitate antisocial and aggressive behavior in adolescents. For this reason, we compared a sample of juvenile offenders, who have committed offenses with legal consequences, with two other samples of different socio-economic characteristics (a community sample and a sample at risk of social exclusion). The results do not fully sustain the differences expected between the three samples in personality traits and intellectual abilities. In fact, the sample at risk of social exclusion and the juvenile offenders sample generally have a common profile in terms of the Big Five personality traits, impulsiveness and physical, verbal, and indirect aggressiveness. The only exception is the trait Conscientiousness: the juvenile offenders have obtained higher scores than the sample at risk of social exclusion, contrary to what was expected. This result may be explained by the fact that the juvenile offenders participate in psychoeducational interventions designed to raise awareness about the consequences of their actions and their responsibility for them. Likewise, the sample at risk of social exclusion and the sample of young offenders generally have a common profile in intelligence because they have obtained the same results on the subscales that assess fluid and crystallized intelligence, with the exception of the subscale Number of PMA.

As expected, the community sample's profile is different from that of the other two samples. In fact, this sample has lower levels of Extraversion and higher levels of Openness to experience. Previous studies have also shown higher levels of Extraversion and lower levels of Openness to experience in adolescents with antisocial behavior (e.g., John et al., 1994; Jin et al., 2016). The results of the current study support Eysenck's theory of criminal personality, according to which the characteristics of extraverted individuals (low levels of self-control, greater need of stimulation from the environment, poor classical conditioning, and lower learning from punishment) facilitate criminal behavior, especially in adolescence, when there is a greater need to take risks and experience new sensations (Eysenck and Gudjonsson, 1989). The trait Openness to experience is related to imagination, curiosity, interest in culture, and art, etc., and the individuals in the samples at risk of social exclusion and juvenile justice may have been raised in more marginal contexts that do not stimulate and value these experiences. We have found no significant differences for Agreeableness and Emotional stability, contrary to what was expected. This result could also be explained by the psychoeducational interventions carried out in the sample of young offenders.

The community sample also differs from the other two samples in their levels of aggressiveness. In fact, the sample of juvenile offenders has higher levels of physical aggressiveness than the community sample, which is congruent with previous studies (e.g., Tremblay, 2003). The sample at risk of social exclusion also has higher levels of physical aggressiveness than the community sample, and higher levels of indirect aggressiveness. These adolescents may be more aggressive because they consider that it is a useful strategy in social interactions (Crick and Dodge, 1994). Moreover, the socioeconomic characteristics of these adolescents may also explain this aggressiveness, because several studies show a relationship between physical aggression and belonging to families with low incomes, having mothers with a low educational level and dysfunctional parental styles (Haapasalo and Tremblay, 1994; Côté et al., 2006). Some evidence also suggests a relationship between indirect aggressiveness and belonging to a dysfunctional family (Pagani et al., 2010). The community sample has lower levels of motor impulsiveness than the other samples. This result was also expected because impulsiveness is closely related to aggressiveness (Vigil-Colet et al., 2008). Furthermore, previous studies also show the relevance of impulsivity to juvenile delinquency (Peach and Gaultney, 2013; Mann et al., 2017) because these adolescents are more likely to act on the spur of the moment, without thinking about the consequences, and using strategies that are not appropriate to the situation.

The community sample is also different from the other two samples in terms of intelligence. In fact, the community sample obtained higher scores on most fluid and crystallized intelligence measures, and also on the G factor. Farrington and West (1990) found a significant negative correlation between intelligence and delinquency, which is congruent with the differences we have found in the current study between the community sample and the sample of juvenile offenders. Moreover, the higher scores on most intelligence measures obtained by the community sample suggest that intelligence is a variable that may play a relevant role in the psychosocial adaptation capacity of an individual, as Huepe et al. (2011) found in their study. In fact, a low intellectual capacity may involve a deficit in prosocial behavior in the medium and long term (Herrnstein and Murray, 1994). Moreover, the lack of strategies for solving problems, such as verbal abilities, is linked to disadaptative behaviors such as antisocial behavior (Garaigordobil, 2004), aggressive behavior and, specially, indirect aggression (Duran-Bonavila et al., 2017). However, in the current study we have not found significant differences in the subscale Vocabulary of PMA, although there are significant differences in other measures of crystallized intelligence. The sample at risk of social exclusion may have some characteristics that affect the development of crystallized intelligence, which is related to learning processes, such as fewer educational opportunities, less stimulation during childhood and an inappropriate family context.

Therefore, these results show that the sample at risk of social exclusion and the sample of juvenile offenders have the same profile in these variables. However, although both samples have similar individual characteristics of personality and intelligence, there must be some risk and protective factors that lead to antisocial behavior in the sample of juvenile offenders, who have committed offenses with legal consequences. The results found with PRA-t and SAVRY provide insight into the key variables that explain the differences between at-risk and delinquent samples and facilitate antisocial behavior. The results show that the sample of juvenile offenders has higher levels of proactive aggressiveness. In fact, several studies show that this kind of aggressiveness is linked to psychopathic features such as callousness and lack of empathy (e.g., Barry et al., 2007; Fite et al., 2010) because it is premeditated aggressive behavior carried out in a “cold-blooded” manner with instrumental, reward-focused goals. In contrast to reactive aggression, this is not emotional and defensive behavior. Other studies have linked proactive aggression with severe forms of antisocial behavior in adolescence, including delinquency (Fite et al., 2010).

There are also significant differences between these two samples in several of SAVRY's risk and protective factors. More specifically, juvenile offenders have a greater history of violence and previous non-violent offending, and were initiated early into violence. In fact, according to Stattin and Magnusson (1996), early initiation of violence is related to higher rates of offending and more serious offenses in adolescence, and a greater persistence of violence from adolescence into adulthood. These adolescents also have higher levels in two family risk factors: early caregiver disruption and poor parental management. These results are congruent with previous studies that show that family dysfunction is associated with higher levels of antisocial behavior and aggressiveness in adolescence (Kennedy et al., 2010; Pagani et al., 2010). In fact, inadequate parental rearing styles, such as the lack of child supervision, inconsistent discipline, or excessive permissiveness are predictors of delinquency in adolescence. The results show the relevance of the environment because juvenile offenders also have higher levels of peer delinquency and community disorganization. Therefore, living in disorganized communities (with high perceived rates of crime, drug sales, gangs, or poor housing) and having delinquent friends may provide more criminal opportunities, which increases the risk of engaging in antisocial behaviors. In fact, the results found on the protective factors of SAVRY also show the relevance of environmental factors because there is a higher percentage of juvenile offenders that do not have strong social support. In other words, in comparison with the sample at risk of social exclusion, they do not have a network of individuals (peer-aged or adult) who provide emotional support and specific assistance in times of distress and need.

As expected, the sample of juvenile offenders generally has low empathy and remorse, characteristics linked to callousness, negative attitudes and a lack of commitment to school, which is congruent with previous studies (e.g., Barry et al., 2007; Fite et al., 2010).

To sum up, the results of this study emphasize the relevance of contextual variables such as the lack of social ties, having peers who commit offenses or dysfunctional relationships with parents, which together with certain individual variables, such as proactive aggression, physical aggression, extraversion, or motor impulsivity, as well as lower scores in intelligence measures, facilitate delinquency, and antisocial behavior. This study, of course, also has some limitations. The administration of the SAVRY questionnaire requires a brief specific training session that the professionals working with juvenile offenders have received, but the professionals working with adolescents at risk of social exclusion may not have. Although we provided information to these professionals to help them with the administration, it is likely that the whole process was easier for juvenile justice professionals. It would also be interesting to know which protective and risk factors are relevant to adolescents at high risk, and whose guardianship has been taken over by the Government. For this reason, further studies should be done that include these kinds of adolescent. Moreover, further studies should also assess other variables that may affect young offenders, such as criminal opportunity, and not only the individual propensity to commit offenses, because opportunity factors are also relevant to the prediction of antisocial behaviors (Beauregard et al., 2007).

## Author contributions

SD carried out the literature research, collected most of the data, and contacted most centers (shared education units, open centers, training and job programs, open intervention programs, and juvenile offenders' education centers). AV formulated the research question, was responsible for the statistical design of the study, carried out the statistical analyses, supervised and critically revised the research, and provided the final approval of the version to be published. SC contributed to the collection of data and contacted some centers. FM contributed to the design of the study and the collection of data, supervised and critically revised the research, wrote the article, and approved the final version to be published.

### Conflict of interest statement

The authors declare that the research was conducted in the absence of any commercial or financial relationships that could be construed as a potential conflict of interest.

## Footnotes

^1^This unit and the others in this paragraph are the units that are part of the Spanish system for compensating social and educational deficits in juveniles at risk of social exclusion.

^2^These are measures of criminal responsibility for minors. In the open programs the young people are not deprived of their freedom while in the education centers they are.
